# Identification and Genetic Characterization of a Novel Respirovirus in Alpine Chamois (*Rupicapra rupicapra rupicapra*)

**DOI:** 10.3390/ani10040704

**Published:** 2020-04-17

**Authors:** Camilla Luzzago, Erika Ebranati, Antonio Lavazza, Martina Besozzi, Gianguglielmo Zehender, Paolo Lanfranchi, Stefania Lauzi

**Affiliations:** 1 Department of Veterinary Medicine, Coordinated Research Center “EpiSoMI”, University of Milan, Via dell’Università 6, 26900 Lodi, Italy; 2 L. Sacco Department of Biomedical and Clinical Sciences, Coordinated Research Center “EpiSoMI”, University of Milan, Via G. B. Grassi 74, 20157 Milan, Italy; erika.ebranati@unimi.it (E.E.); gianguglielmo.zehender@unimi.it (G.Z.); 3 Istituto Zooprofilattico Sperimentale della Lombardia e dell’Emilia Romagna, Via Bianchi 9, 25124 Brescia, Italy; antonio.lavazza@izsler.it; 4 Department of Veterinary Medicine, University of Milan, Via dell’Università 6, 26900 Lodi, Italy; m.besozzi@alpvet.it (M.B.); paolo.lanfranchi@unimi.it (P.L.); stefania.lauzi@unimi.it (S.L.); 5 Studio Associato AlpVet, Piazza Venzaghi, 21052 Busto Arsizio, Italy

**Keywords:** novel respirovirus, *Paramixoviridae*, chamois, wild ruminants, pneumonia

## Abstract

**Simple Summary:**

The Alpine chamois is a free-living wild ruminant distributed across the mountainous areas of France, Italy, Switzerland, Liechtenstein, Germany, Austria, Slovenia and Croatia. This wide distribution favours interactions with other wild ruminants and livestock, implying the risk of cross-transmission of pathogens. Due to the impact of lung diseases on chamois populations, the investigation of respiratory pathogens is important for wildlife conservation and for the understanding of infection transmission at the livestock–wildlife interface. In the present study, a novel respirovirus was isolated from a chamois with lung lesions and characterized by full-genome sequence and phylogeny. The genome characterization of this novel virus revealed similarities to domestic ruminant respiroviruses, mainly of caprine and ovine origin. Overall, phylogenetic analyses indicated that the chamois virus is distinct from already defined species and suggested that it is a putative novel member of the genus *Respirovirus*. The present investigation contributes to the knowledge of respiratory infections in wild ruminants and raises questions on the epidemiological link between chamois and other animal species.

**Abstract:**

The *Respirovirus* genus, family *Paramamixoviridae*, includes respiratory viral pathogens. Here we report the identification and genetic characterization of a respirovirus in an Alpine chamois showing interstitial pneumonia associated with catarrhal bronchopneumonia. The full-genome characterization of this respirovirus, named ChamoisRV/IT2014, revealed low similarities to caprine respirovirus (77.1%), bovine respirovirus (74.5%) and human respirovirus (72.0%). The phylogenetic analyses based on the full-length genome sequence of the novel isolate and reference respirovirus strains showed that ChamoisRV/IT2014 clustered with caprine respirovirus but formed a separate branch. The phylogenetic tree topology of complete large protein amino acid sequences, representing the current species demarcation criterion for Respirovirus genus, showed a 0.05 branch length of ChamoisRV/IT2014 sequence between the nearest node and the tip of the branch, suggesting that this virus belongs to a novel species. This new isolate in a new host species raises several questions to be addressed on the epidemiological role of chamois and the risks of cross-transmission between wild ruminants and livestock.

## 1. Introduction

In recent years, novel respiroviruses have been identified in goats suffering respiratory syndrome in China [1,2] and in the Sri Lankan Giant squirrel introduced in Germany [3]. To date, the *Respirovirus* genus in the family *Paramyxoviridae* includes six species: human respirovirus 1 and 3 (HRV1, HRV3; formerly human parainfluenza virus 1 and 3), porcine respirovirus 1 (PRV3, formerly porcine parainfluenza virus 1), bovine respirovirus 3 (BRV3, formerly bovine parainfluenza virus 3), caprine respirovirus 3 (CRV3, formerly caprine parainfluenza virus 3) and murine respirovirus (MRV, formerly Sendai virus), as recently revised [4].

CRV3 (formerly CPIV3) was first identified in goats [2] and recently also in sheep in China [5]. To date, CRV3 has not been reported in other countries.

BRV3 (formerly BPIV3) is a worldwide cattle respiratory pathogen involved in bovine respiratory disease (BRD) [6], a multifactorial disease for which viral pathogens have a prominent role as primary agents [7]. Serological evidence of BRV3 infection has been reported in several wild ruminant species, including Alpine chamois in Italy [8], bighorn sheep [9] and white-tailed deer [10] in North America. Due to the impact of the pneumonia complex on chamois populations, investigation on respiratory pathogens is of considerable interest [11].

The Alpine chamois is the most abundant subspecies of *Rupicapra* spp. with nearly 500,000 individuals distributed among France, Italy, Switzerland, Liechtenstein, Germany, Austria, Slovenia and Croatia [12]. This expansion favours the interactions between livestock and wild ruminants, implying an increased risk of cross-transmission of pathogens. Indeed, some of the major outbreaks reported in chamois in Europe have been triggered by pathogens initially cross-transmitted at the interface with livestock [13].

Considering the need to widen the knowledge on respiratory infection of chamois, in the present study we report a novel respirovirus isolated from chamois with lung lesions that was characterized by the full-genome sequence and phylogeny.

## 2. Materials and Methods

### 2.1. Study Area and Sampling

The study area was located in the north-western Italian Alps (45°56′ N, 8°34′ E) where chamois, roe deer (*Capreolus capreolus*), red deer (*Cervus elaphus*) and ibex (*Capra ibex*) are present. Furthermore, cattle and small ruminant herds share alpine pastures during summer season. Chamois, roe deer and red deer are legally selectively hunted in accordance with Italian law (N. 157 of 11/02/1992) and inspected by veterinarians at the wildlife control centres of the area. In autumn 2014, coughing chamois were reported by hunters. In September–October 2014, 191 chamois were legally selectively hunted (11 kids, 61 yearlings, 119 >2 years old). To collect data on respiratory infections, lungs were available only for 14 yearlings, both with and without macroscopic lesions, and for 7 adults with macroscopic lesions. Lung samples were collected and stored at −20 °C for 1–2 weeks in the control centres facility and subsequently transferred to the laboratory at −80 °C until further processing. Blood samples, collected from major blood vessels or heart clots, were also available for 1 kid, 3 yearlings and 7 adults. Samples were centrifuged and the serum was stored at −20 °C until further processing.

### 2.2. Viral Isolation

Tissue homogenates (approx. 10% *w/v*) of lungs were inoculated in 24-well plates in sub-confluent monolayers of Madin–Darby bovine kidney cells (MDBK ATCC CCL-22). MDBK cells were maintained in minimal essential medium (MEM) with 1% L-glutamine 200 mM, 100 U/mL penicillin, 100 µg/mL streptomycin, 2.5 µg/mL fungizone and 10% of fetal bovine serum (FBS), free of antibodies to bovine herpes virus-1, bovine respiratory syncytial virus, bovine parainfluenza virus 3 and free of both virus and antibodies to bovine viral diarrhea virus. The inoculated plates were incubated at 37 °C in 5% CO_2_ and after a 1–2 h adsorption period the cell cultures were rinsed and maintenance medium was added. The cell cultures were observed daily for cytopathic effect (CPE) for 6 days. Two blind passages were made if no CPE was observed; the cell cultures were scraped and vigorously mixed with culture medium and used for inoculation of fresh monolayers.

### 2.3. RT-PCR

Viral RNA was extracted from lysates of cell cultures showing CPE using TRIZOL^®^ LS reagent (Invitrogen, Carlsbad, CA, USA), according to the manufacturer’s instructions. The RNA was retrotranscribed using a QIAamp One-For-All Nucleic Acid kit (Qiagen, Mississauga, ON, Canada) and cDNA was subject to PCR according to previous published protocols for bovine and ovine respiratory syncytial virus [14,15], pan-coronavirus [16], pestivirus [17,18] and mammalian orthoreoviruses [19].

### 2.4. Bacteriological Examination

Bacteriological examination of tissue samples was performed on blood–agar plates under aerobic and microaerophilic conditions. Plates were incubated at 37 ± 2 °C for 24 and 48 h.

### 2.5. Negative Staining Electron Microscopy

Due to negative results from specific PCR panels, the supernatant fluid from cell culture showing CPE was submitted to negative staining electron microscopy (nsEM) using the Airfuge method [20]. Samples were ultracentrifuged (Airfuge, Beckman Coulter Inc. Life Sciences, Indianapolis, Indiana, USA) for 15 min at 82,000× *g* using a rotor holding six 175-μL test tubes in which specific adapters for 3 mm carbon-coated Formvar copper grids were placed. Grids were stained with 2% NaPT, pH 6.8 for 1.5 min and examined at 13,500–43,000x by using a Tecnai G2 Spirit Biotwin transmission electron microscope (FEI, Hillsboro, Oregon, OR, USA) operating at 85 kV and at 20,500–43,000× for at least 15 min before being considered negative. Attempts to identify the observed viral particles were based on their morphological characteristics.

### 2.6. Whole Genome Sequence

The whole genome was amplified by using the sequence-independent single-primer amplification (SISPA method) and sequenced by Next Generation Sequencing (NGS) method [21]. In detail, RNA was extracted from the cell culture supernatant by the QIAamp viral RNA mini Kit and the automatic QIACUBE robot (Qiagen, GmbH, Hilden, Germany). Extracted RNA was reverse-transcribed using the random primer FR26RV-N (5′GCCGGAGCTCTGCAGATATCNNNNNN3′) at a concentration of 10 µM. Viral cDNA was denatured at 94 °C for three min and chilled on ice for two min. Five units of Klenow fragment (New England Biolabs, Ipswich, MA, USA) were directly added to the reaction to perform the second strand cDNA synthesis. The incubation was carried out at 37 °C for one h and at 75 °C for 10 min.

Following this step, 5 µL of double-stranded DNA was added to a PCR master mix containing 5 µL of 10× AccuPrime PCR buffer I; 0.2 µL of AccuPrime Taq DNA Polymerase, high fidelity; 4 µL of 10 µM FR20RV (5′GCCGGAGCTCTGCAGATATC3′) and 35.8 µL of water. The incubation was performed under the following thermal conditions: 94 °C for two min, followed by 40 cycles of 94 °C for 30 s, 55 °C for one min and 68 °C for three min. The PCR product was purified and quantified using a TECAN plate reader. The sample was diluted to an initial concentration of 0.2 ng/µL in accordance with the Illumina protocol, and 1 ng was used for the library preparation (Nextera XT sample preparation Kit, Illumina Inc., San Diego, CA, USA).

Genomic libraries were sequenced on the Illumina MiSeq platform (Illumina, Inc.) with 2 × 151 base pairs paired-end runs. Finally, the obtained reads were evaluated for sequence quality and read-pair length using FastQC ver. 0.11.5. The reads were assembled using Geneious software v. 11.1.5 (Biomatters, New Zealand) and re-sequencing analysis was performed with the complete genomes retrieved from Genbank. The full-length genome sequence of the chamois isolate was deposited into GenBank (accession number MT180123).

### 2.7. Phylogenetic Analysis

The novel complete genome and nucleotide sequences of genome segments were compared for similarity against all sequences available from GenBank using Basic Local Alignment Search Tool (BLAST) analysis. Multiple sequence alignments of the novel complete genome were performed with reference sequences of genus *Respirovirus* using Clustal W. The phylogeny of the complete genome was estimated by the neighbor-joining algorithm (NJ) and the maximum likelihood (ML) method (MEGA version X), with 1000 replicates. Sequence identity of the nucleotide and deduced amino acid sequences of genome segments were calculated against reference sequences of genus *Respirovirus* using Bioedit software version 7.0 [22]. Analysis of DNA polymorphisms and synonymous or nonsynonymous sites were performed using DnaSP software [23]. Estimations of positive selection sites were performed using Datamonkey [24]. The non-synonymous and synonymous substitution rates were calculated the following methods: random effects likelihood (REL); fixed effects likelihood (FEL); a single likelihood ancestor counting (SLAC) and branch-site Unrestricted Statistical Test for Episodic Diversification (BUSTE).

### 2.8. Serological Screening

Sera were tested by virus neutralization tests (VNT) against the novel chamois strain, bovine respiratory syncytial virus (BRSV Strain 375) and bovine viral diarrhea virus (BVDV strain NADL). Two-fold serial dilutions of heat-inactivated serum in duplicate were mixed with equal volumes of virus containing 100 TCID50. The VNT was performed on MDBK cells, maintained in MEM supplemented as previously described and with 10% of FBS. The plates were incubated at 37 °C with 5% of CO_2_ for 96 h for Chamois strain and BVDV, and for 5 days for BRSV. VN titer was defined as the highest serum dilution able to inhibit virus replication. A titer > 4 was defined as positive.

## 3. Results

**Figure 1:**
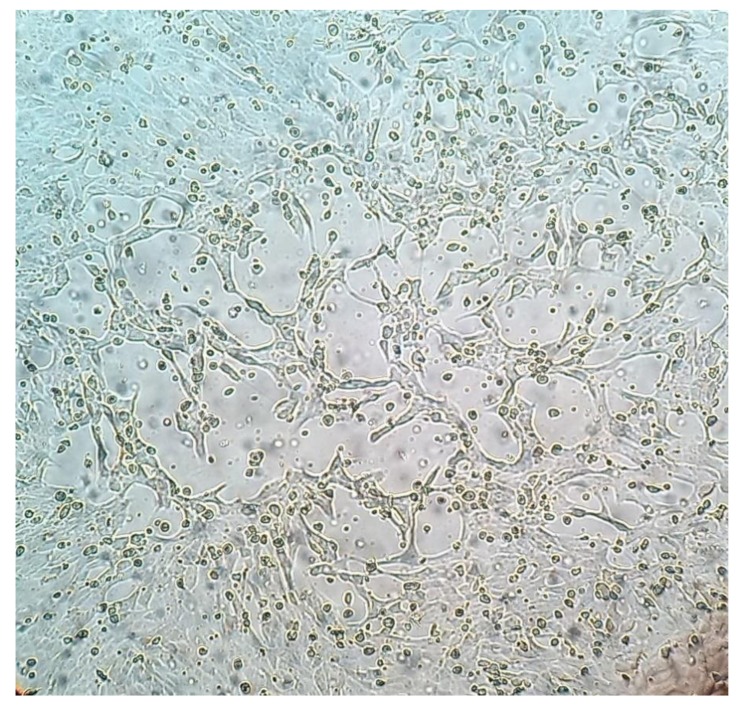
Madin–Darby bovine kidney (MDBK) cells infected with ChamoisRV/IT2014 strain showing lytic cytopathic effect (CPE) at 72 h post infection.

At the control center, macroscopic lesions of interstitial pneumonia in association with catarrhal bronchopneumonia were observed in four adult chamois (two males and two females) and interstitial pneumonia was observed in five yearlings (four males and one female) and three adult females. Relevant bacterial pathogens known to cause respiratory disease were not identified by bacteriological examination of the lungs. Evidence of virus growth was detected only from the lung of a 13-year-old hunted male chamois with interstitial pneumonia associated with catarrhal bronchopneumonia. The CPE was observed at the first passage of the cell lines from 72 h post infection (Figure 1).

**Figure 2:**
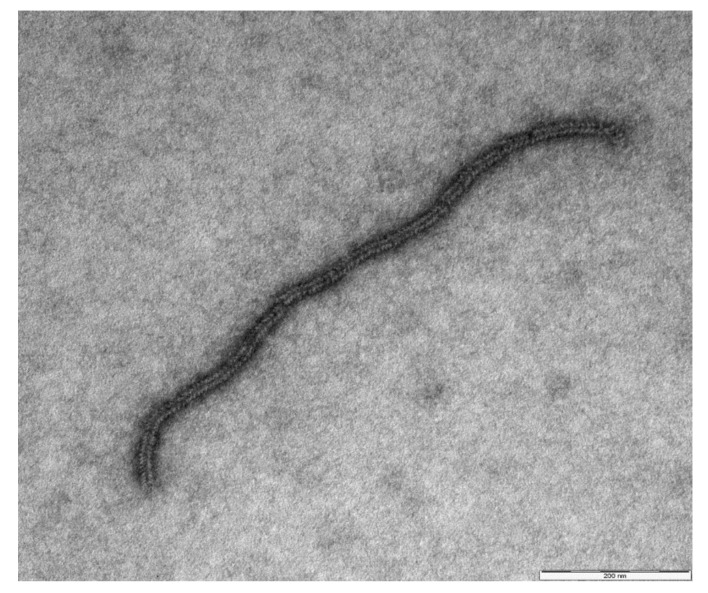
Long fragment of free nucleocapsid chains originating from the disintegration of respirovirus particles in the supernatant of a CPE-positive MDBK cell culture infected with ChamoisRV/IT2014. Negative staining electron microscopy (nsEM), NaPT 2%. Bar = 200 nm.

The CPE–positive sample was negative to specific PCR panels. The supernatant of the infected cell cultures preliminarily observed by the nsEM revealed the presence of virus particles with morphological features indicative of the paramyxovirus family. In particular, in addition to some intact virions, several disintegrating particles with free nucleocapsid chains were observed (Figure 2). Subsequently, a complete viral genome was efficiently obtained starting from the cell culture supernatant.

BLAST analysis of the whole genome of the chamois virus, named ChamoisRV/IT2014, revealed CRV3 as the most similar sequence (accession number MK091103 80.1%). The whole genome of ChamoisRV/IT2014 showed 77.1% nucleotide identity with CRV3 reference strain JS2013, 74.5% with BRV3 genotype A (BRV3a) strain TVMDL60, 74.2% with BRV3 genotype B strain Q5592, 73.9% with BRV3 genotype C strain SD0835 and 72.0% with HRV3 strain AUS/5/2007.

**Figure 3:**
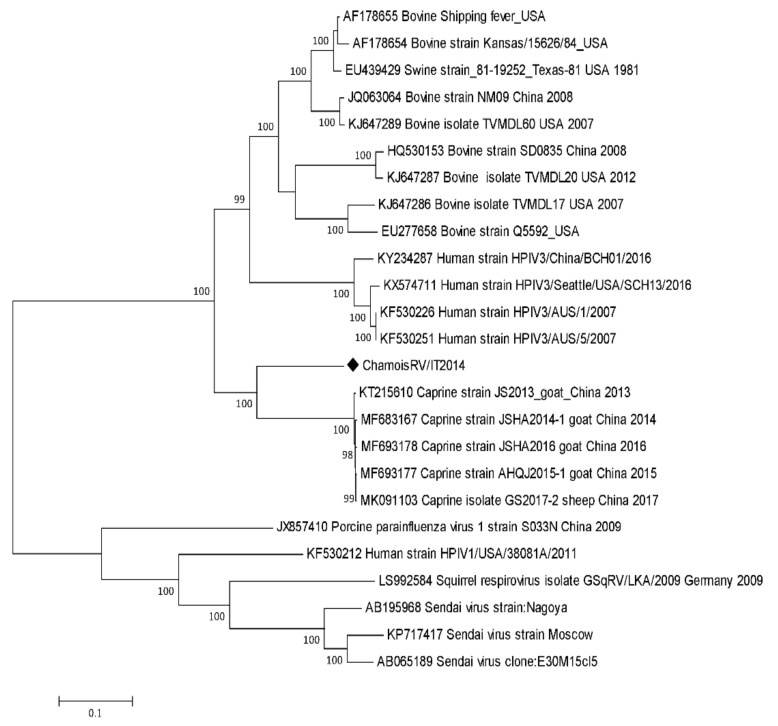
Phylogenetic tree based on the complete genome of ChamoisRV/IT2014 and a selection of representative sequences of the *Respirovirus* genus. Molecular evolutionary genetic analyses were performed with MEGA X software using the maximum likelihood (ML) method based on the Tamura–Nei model. Bootstrap values > 70% are shown. Published sequences and references are identified by the GenBank accession number [25]. The black diamond indicates the novel sequence from chamois obtained in the present study.

The phylogenetic tree with other respirovirus strains, including publicly available sequences of BRV3, CRV3, HRV1–3, PRV1 and MRV, showed that ChamoisRV/IT2014 clustered with RV3 (Figure 3) and formed a significant clade with CRV3 [2] using the ML method. Similar results were obtained using NJ.

**Figure 4:**
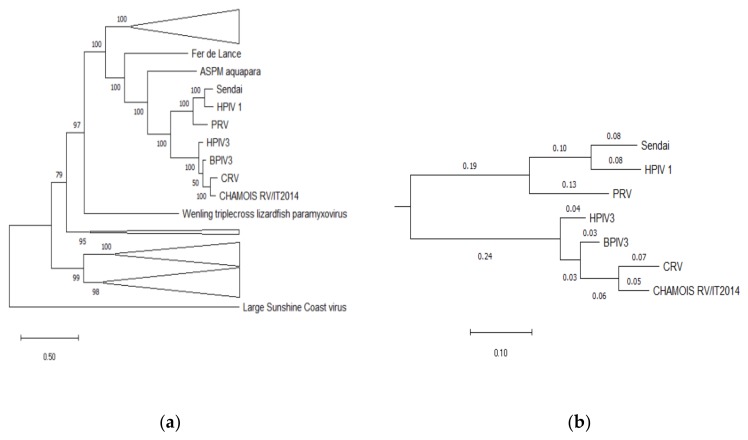
Phylogenetic analysis of complete large (L) protein amino acid sequences of members of the family *Paramyxoviridae*. (**a**) Complete L protein amino acid sequences were aligned by Clustal W with gap generation penalties of 5 and extension penalties of 1 in both multiple and pairwise alignments. The evolutionary history was inferred by using the Maximum Likelihood method and JTT matrix-based model. The tree is drawn to scale, with branch lengths measured in the number of substitutions per site. Bootstrap values > 70% are shown and clades of other than the respirovirus genus are compressed (**b**) The respirovirus genus subtree is expanded, showing branch lengths measured in the number of substitutions per site. Evolutionary analyses were conducted in MEGA X software. Sequence alignment is downloaded from [26].

Pairwise comparisons of the nucleotide sequence of coding regions and corresponding deduced amino acid sequences of ChamoisRV/IT2014 with reference strains of CRV3, BRV3 and HRV3 genotypes are reported in Table 1. The ChamoisRV/IT2014 nucleotide sequence of coding regions showed a slightly higher identity with CRV3 compared to BRV3, reaching values ranging from 78.2% for hemagglutinin-neuraminidase (HN) to 81.9% for the nucleoprotein (N) gene. The only exception was phosphoprotein (P) that shared a similar identity with both CRV3 (72.2%) and BRV3 (72.2%–73.1%). The matrix (M), large (L) and N protein sequences showed higher amino acid identity values compared to the other coding regions, sharing with CRV3 the highest values of 91.5%, 89.2% and 89.1%, respectively. The P protein sequence showed the lowest amino acid identity values compared to the other coding regions, ranging from 53.0% with HRV3 to 59.2% with CRV3. The phylogenetic tree topology of complete L protein amino acid sequences of members of the family *Paramyxoviridae* showed a 0.05 branch length of ChamoisRV/IT2014 sequence between the nearest node and the tip of the branch (Figure 4).

A highly conserved motif, located near the middle of the N proteins and thought to be essential in N–N self-assembly and the N–RNA interaction process, was found in the central domain of the N proteins from the chamois virus, as ^323^FAPGNYPALWSYAM^336^, and was also observed in CRV3 [2]. The HN protein contained the conserved binding site sequence, ^254^NRKSCS^259^ [26], suggesting a neuraminic acid containing cellular receptor. The results of the analysis of DNA polymorphisms and synonymous or nonsynonymous sites are reported in the Supplementary data (Table S1). Concerning the analysis of the estimation of positive selection sites, there was no evidence that any sites have experienced diversifying selection.

Concerning the serological analysis (Table 2), four animals were positive to ChamoisRV/IT2014 strain, with VN titers ranging from 8 to 256; two chamois demonstrated seropositivities to BRSV, including one chamois with lung lesions that showed seropositivity also to ChamoisRV/IT2014 strain. All sera were negative for BVDV.

## 4. Discussion

We report the isolation, identification and genetic characterization of a novel RV3, found in a hunted, free-living Alpine chamois showing interstitial pneumonia associated with catarrhal bronchopneumonia. The finding of the novel RV3 being limited to one animal only could be related to a likely short–phase of virus replication in the lungs, and direct detection was thus restricted. Moreover, virus isolation is a diagnostic technique that requires a virus that is able to replicate in cell culture, therefore the sensitivity could be limited. The use of pan-paramyxoviridae primers previously published, especially universal primers for respiroviruses [27], as well as the set-up of a specific RT-PCR have to be considered in respiratory pathogen panels to improve the diagnostic sensitivity and confirm the presence of the novel RV3 RNA in lung samples of chamois. The evidence of seropositive chamois, showing VN antibodies against ChamoisRV/IT2014, supports the circulation of this infection in the study area. This needs to be investigated to determine the real diffusion of this novel agent in chamois population and establish its epidemiological role.

The absence of other respiratory agents in the lungs of the RV3-positive and other chamois supports the hypothesis that the novel virus in chamois may be the etiological agent of the lung lesions that could either be interstitial pneumonia and/or catarrhal bronchopneumonia. Nevertheless, the negative results for other viral or bacterial respiratory pathogens could be biased by the storage condition of lung samples (−20 °C) and by an analytical protocol focused on viral isolation. The phylogenetic analyses based on the full-length genome sequence of the novel isolate and reference respirovirus strains showed that ChamoisRV/IT2014 significantly clustered with CRV3 but formed a separated branch. Despite this, we generated a comprehensive dataset of animal RV3 publicly available from GenBank; there were relatively few whole genome sequences available, and no other RV3 sequence clustered with ChamoisRV/IT2014. The nucleotide identity for the ChamoisRV/IT2014 complete genome with respirovirus reference strains, including CRV3, was 80% or below, revealing nucleotide differences higher than the ones observed among genotypes and members of *Respirovirus* genus [2,28]. The comparison of complete L protein amino acid sequences, representing the current species demarcation criterion for *Respirovirus* genus [26], shows that ChamoisRV/IT2014 possesses sequence differences from the other respirovirus species. Moreover, although the phylogenetic analysis of ChamoisRV/IT2014 based on M, HN and F genes, used as targets for genetic typing of RV3 [5], confirms the highest nucleotide identity with CRV3, it shows low nucleotide identity for all three genes.

Overall, the results of the full genome phylogenetic tree, sequence identities with other respirovirus and species demarcation criterion for the *Respirovirus* genus, indicate that ChamoisRV/IT2014 is distinct from already defined species and suggest that it is a putative novel member of the genus *Respirovirus*.

This new isolate in a new host species raises several questions to be addressed. Studies on the epidemiological role of chamois, the risks of cross-transmission between wild ruminant and livestock and the evolution mechanisms, including genomic changes driven by the adaptation of viruses to new hosts, are suggested. The new powerful methods for genomic characterization will contribute to increase the availability of whole genome sequences to deepen the understanding of the origin and evolution of respiroviruses.

## Figures and Tables

**Table 1:** Nucleotide and amino acid identities for each gene segment of the novel respirovirus sequence from chamois compared to respirovirus reference strains from GenBank.

Coding Regions	Identity %									
ChamoisRV/IT2014 Gene Region	CRV3 JS2013		BRV3a TVMDL60		BRV3b Q5592		BRV3c SD0835		HRV3 AUS/5/2007	
	**nt ^1^**	**aa ^2^**	**nt**	**aa**	**nt**	**aa**	**nt**	**aa**	**nt**	**aa**
Nucleoprotein (N)	81.9	89.1	80.1	88.0	80.3	87.4	78.4	85.1	78.2	81.6
Phosphoprotein (P)	72.2	59.2	72.7	56.6	73.1	57.4	72.2	56.0	69.8	53.0
Matrix protein (M)	82.4	91.5	78.4	86.7	79.1	87.8	78.6	87.8	78.4	86.7
Fusion protein (F)	79.2	84.1	77.1	80.7	76.9	81.3	75.7	79.4	73.3	75.5
Hemagglutinin/neuraminidase (HN)	78.2	83.7	75.0	77.2	75.8	77.9	73.8	75.0	71.6	69.7
Large protein (L)	81.1	89.2	80.3	87.9	79.4	87.6	79.4	86.9	78.4	85.3

**^1^** nucleotide **^2^** amino acid.

**Table 2:** Virus neutralization titers of hunted chamois sera against the chamois strain from this study, bovine respiratory syncytial virus and bovine viral diarrhea virus.

Chamois Id.	Collection Date	Age in Years	Gender	Chamois RV/IT2014	BRSV	BVDV
57311 ^a^	28-9-2014	1	M	64	64	2
100971	28-9-2014	2	F	2	23	2
81720	28-9-2014	3	F	8	2	2
20781	1-10-2014	4	M	2	2	2
23204	1-10-2014	1	F	2	2	2
75777 ^b^	1-10-2014	11	F	2	2	2
83284	1-10-2014	4	F	2	2	2
23504	5-10-2014	6	M	32	2	2
39766	8-10-2014	<1	M	2	2	2
83469	19-10-2014	1	M	256	2	2
62516	22-10-2014	13	F	2	2	2

^a^ interstitial pneumonia, negative to virus isolation; ^b^ interstitial pneumonia and catarrhal bronchopneumonia, negative to virus isolation.

## Supplementary Materials

The following is available online at https://www.mdpi.com/2076-2615/10/4/704/s1, Table S1: Analysis of DNA polymorphisms and synonymous or nonsynonymous sites of ChamoisRV/IT2014 and selected respirovirus sequences from Genbank reported in Table 1.
