# Supplementary material for: Identification and Genetic Characterization of a Novel Respirovirus in Alpine Chamois (Rupicapra rupicapra rupicapra)
Source: Animals (Basel). 2020 Apr 17;10(4):704. doi: 10.3390/ani10040704 (PMC7222786; doi:10.3390/ani10040704)
Supplement: Supplementary file 1 [file animals-10-00704-s001.pdf]

animals

Supplementary Materials: Identification and Genetic Characterization of a Novel Respirovirus in Alpine Chamois (R*upicapra rupicapra rupicapra*)

Camilla Luzzago [1,*], Erika Ebranati [2], Antonio Lavazza [3], Martina Besozzi [4], Gianguglielmo Zehender [2], Paolo Lanfranchi [5] and Stefania Lauzi [5]

[1] Department of Veterinary Medicine, University of Milan, Coordinated Research Center "EpiSoMI", Via dell'Università 6, 26900 Lodi, Italy
[2] L. Sacco Department of Biomedical and Clinical Sciences, Coordinated Research Center "EpiSoMI", University of Milan, Via G. B. Grassi 74, 20157 Milan, Italy; erika.ebranati@unimi.it (E.E.); gianguglielmo.zehender@unimi.it (G.Z.)
[3] Istituto Zooprofilattico Sperimentale della Lombardia e dell'Emilia Romagna, Via Bianchi 9, 25124 Brescia, Italy; antonio.lavazza@izsler.it
[4] Department of Veterinary Medicine, University of Milan, Via dell'Università 6, 26900 Lodi, Italy; Studio Associato AlpVet, 21052 Busto Arsizio, Italy; m.besozzi@alpvet.it
[5] Department of Veterinary Medicine, University of Milan, Via dell'Università 6, 26900 Lodi, Italy; paolo.lanfranchi@unimi.it (P.L.); stefania.lauzi@unimi.it (S.L.)
* Correspondence: camilla.luzzago@unimi.it; Tel.: +39-02-503-18068-34527

Table S1. Analysis of DNA polymorphisms and synonymous or nonsynonymous sites of ChamoisRV/IT2014 and selected respirovirus sequences from Genbank reported in table 1.

ChamoisRV/IT2014 Gene Region	S[1]	π[2]	Syn Dif Min–Max Range[3]	NSynDif Min–Max Range[4]	NSyn fraction Min–Max Range[5]
Nucleoprotein (N)	621	0.20724	221.00–241.50	74.00–137.67	0.0645–0.1248
Phosphoprotein (P)	869	0.26150	158.83–195.58	234.17–325.42	0.2025–0.2984
Matrix protein (M)	406	0.20069	153.00–173.83	33.00–57.33	0.0420–0.0748
Fusion protein (F)	740	0.23354	236.00–270.17	105.00–184.83	0.0892–0.1654
Hemagglutinin/neuraminidase (HN)	847	0.25515	261.67–283.67	126.33–243.67	0.1023–0.2115
Large protein (L)	2574	0.19355	648.33–712.67	622.67–754.75	0.1286–0.1585

[1] number of polymorphic sites [2] nucleotide diversity [3] number of synonymous difference: ChamoisRV/IT2014 *versus* respirovirus reference strains [4] number of non-synonymous difference: ChamoisRV/IT2014 *versus* respirovirus reference strains [5] non-synonimous difference fraction: ChamoisRV/IT2014 *versus* respirovirus reference strains.
